# Observational Study of Qigong as a Complementary Self-Care Practice at a Tertiary-Care Pain Management Unit

**DOI:** 10.1155/2021/6621069

**Published:** 2021-06-17

**Authors:** Lauren Curry, Meghan Pike, Mary Lynch, Dana Marcon, Jana Sawynok

**Affiliations:** ^1^Faculty of Medicine, Dalhousie University, Halifax, Canada; ^2^Department of Pediatrics, IWK Health Centre, Halifax, Canada; ^3^Departments of Anesthesia, Pain Management & Perioperative Medicine, also Pharmacology, and Psychiatry, Dalhousie University, Halifax, Canada; ^4^Kaizen Coaching, Halifax, Canada; ^5^Departments of Pharmacology, also Anesthesia, Pain Management & Perioperative Medicine, Dalhousie University, Halifax, Canada

## Abstract

Qigong, which can be characterized in many different ways, is offered as a complementary self-care practice at a tertiary-care pain management unit in Halifax, Nova Scotia. This report provides a quantitative and qualitative assessment of two groups engaged in qigong practice in this context as part of two Research in Medicine (RIM) projects (2014-15, 2016-17). It includes assessments of pain, mood, quality of life, sleep, and fatigue, considers outcomes in relation to the amount of practice, and considers whether health attitudes would help determine who might benefit from the practice. There were 43 participants (28 ongoing practitioners, 15 new to qigong). The ongoing practice group in RIM2 had significant benefits over time in pain, mood, quality of life, and fatigue in quantitative scores, but changes were not significant in RIM 1. There were no differences in any measures in those new to qigong. Qualitative comments in core and other domains reflected good or better outcomes in 16 subjects in the ongoing group who practiced consistently. In those who practiced less, results were more variable. In most of those new to qigong, the practice was limited and comments indicate minimal changes. Those engaged in qigong have a stronger internal health locus of control than control subjects. Diligent qigong practice provides multiple health benefits for those with chronic pain, and qualitative assessments are essential for documenting these effects. For those new to qigong, factors needed to effectively engage practice need to be explored further to optimize program delivery. The trial is registered with http://www.clinicaltrials.gov (NCT04279639).

## 1. Introduction

Qigong is a traditional Chinese practice involving specific movements, breathing methods, and meditation and is promoted for health maintenance and improvement. Since the 1980s, and particularly since 2000, there has been considerable interest in qigong as a potential therapeutic modality. Contemporary descriptions of qigong include traditional Chinese exercise [1], therapeutic exercise [2], mindfulness-based exercise [3, 4], mind-body therapy [5], meditative movement [6], and movement-based embodied contemplative practice [7]. Qigong is being examined in multiple health domains and is emerging as a promising complementary practice in several areas (cancer treatment, respiratory disorders, cardiovascular disorders, and movement disorders) [2]. There are also beneficial effects on chronic pain, fatigue and sleep, and mood disorders [1, 3–5].

Fibromyalgia is a chronic pain condition with common comorbidities of sleep and mood disorders [8]. In 2012, we published a controlled trial of qigong for fibromyalgia involving two groups of subjects (immediate and delayed training groups) and reported reproducible and significant benefits in pain, sleep, impact, and physical and mental function [9]. Benefits in all areas were related to the amount of practice during the 24-week trial. Some participants continued with their practice beyond the trial and attained marked health benefits [10]. Further studies on qigong for fibromyalgia at other sites (durations 6–24 weeks) indicated consistent benefits in those who engage in regular (daily or near-daily) practice but inconsistent effects with weekly sessions [11]. As a result of local experiences, qigong classes have been offered as a complementary self-care practice at a local tertiary-care pain management unit.

The present report is an observational study of participants who undertook qigong as a voluntary self-care practice at the pain management unit in Halifax, Nova Scotia. Two groups of individuals were observed (2014-2015, 2016-2017) in the context of Research in Medicine (RIM) projects by two medical students. The trial provided an opportunity to document experiences of those involved in long-term qigong practice over several years (few trials extend beyond 24 weeks) and of those new to the practice (providing insight into knowledge translation from the controlled trial). A mixed-methods approach was used, combining quantitative measures (for pain, mood, quality of life, sleep, and fatigue) and qualitative comments (open-ended comments relating to the same domains). Such approaches provide a more complete reflection of patient experiences, especially with regard to chronic pain [12, 13]. An additional questionnaire assessing health locus of control was included as the practice involves self-care and application care in practice.

The aims of the study were (a) to provide quantitative and qualitative assessments of participant experiences of qigong as a complementary practice in a real world-setting of chronic pain management, (b) to consider outcomes in relation to the amount of practice, and (c) to determine whether attitudes might be helpful for predicting who might benefit from the practice. Six of the cases from this observational trial reporting remarkable outcomes with the long-term practice of qigong have already been published as a case series [14]; the current analysis includes consideration of their quantitative and attitudinal scores as part of the entire group.

## 2. Methods

### 2.1. General

This study represents two observational intervals of those who undertook qigong as a voluntary self-care practice at the Queen Elizabeth II Health Science Centre's Pain Management Unit in Halifax, Nova Scotia, Canada. It was approved by the appropriate Research Ethics Board prior to commencement. In 2012, a published report indicated significant benefits of Chaoyi Fanhuan Qigong (CFQ), a form of qigong available locally (http://www.cfqcanada.com), in those with fibromyalgia in relation to pain and other health areas [9]. Qigong classes have been offered since 2008 when the results of a pilot trial were available. Participants entered the current study based on former experience with the practice in response to pamphlets available in the clinic reception area and by word of mouth. The report summarizes participant experiences undertaken as RIM projects between July 1, 2014 and May 31, 2015 (MP), and July 1, 2016 and May 31, 2017 (LC). The study was registered retrospectively at clinicaltrials.gov (NCT04279639).

### 2.2. Participants

In total, the study involved 43 subjects, with *N* = 29 participating in RIM1 and *N* = 29 in RIM2. The latter involved overlap subjects who continued from RIM1 and some who had prior experience with qigong but had not participated in RIM1. There were 15 subjects who were new to qigong (*N* = 4 in RIM1 and *N* = 11 in RIM2).  Table 1 outlines the components of the trial and identifies specific numbers who participated in each component.  Table 2 presents entered demographics, including gender, age, pain diagnoses, and relevant medical history.

### 2.3. Measures

Consistent with recommendations for chronic pain studies [15], quantitative measures included assessments of pain (BPI, Brief Pain Inventory), mood (POMS, Profile of Mood Scale), sleep (PSQI, Pittsburgh Sleep Quality Index), fatigue (CFS, Chronic Fatigue Scale), and quality of life (SF-12) [9]. In addition, qualitative comments were collected reflecting these domains (pain, sleep, other health areas, quality of life, and medications) via open-ended surveys. As there is literature indicating health benefits are related to the amount of qigong practice within a trial [9, 11, 16], participants completed weekly logs of practice times (minutes/day, days/week). The times at which data collection took place are summarized in Table 1.

In order to assess health attitudes, an additional questionnaire related to Health Locus of Control (HLC) was included. This is a set of 11 questions relating to attitudes towards the degree of control one has over illness [17], which has been used to characterize chronic pain populations [18]. In order to have a comparator group, the HLC questionnaire was also distributed to a sample of those attending the pain management unit who did not undertake qigong practice (July-Aug 2016).

### 2.4. Intervention

Chaoyi Fanhuan Qigong, CFQ (http://www.cfqcanada.com), consists of two levels of instruction. Level 1 consists of 7 movements, with a set consisting of 10 repetitions of movements 1–5 and 5 repetitions of movements 6-7. Each set takes approximately 15 minutes to perform. Level 2 instruction consists of meditative instruction and involves sitting, laying, and standing postures. There is also instruction in additional ancillary exercises. Weekly classes during the 6-week sessions consisted of 120 minutes of instruction and practice. Instruction was delivered by an instructor (DM) with at least 10 years of experience; she had also provided instruction in the randomized controlled trial and was experienced working with those with chronic pain [9]. Participants were encouraged to practice daily for 15–45 minutes initially and then for longer when they became more experienced. Practice times, especially longer ones, consist of a mix of level 1, level 2, and ancillary practices. Sessions were offered six times throughout the year (Table 1), and participants were encouraged to maintain their home practice between sessions.

### 2.5. Analysis

This study involves considerable heterogeneity and multiple viewpoints were required to assess outcomes. Quantitative measures were analysed for each interval using paired *t*-tests to compare outcomes for each 6-week interval to the entry value. For those new to qigong, the entered value is a true baseline (*N* = 4 in RIM1, *N* = 11 in RIM2; total *N* = 15) and this group is considered separately. For those with prior qigong experience, the study entry value only provides an anchor value. Qualitative assessments are presented in tables, categorized as good or variable outcomes, and constitute a narrative approach. The experiences of those new to qigong are presented separately, as the duration of their experience vastly differs from that of ongoing practitioners. HLC measures were compared to a control group (*N* = 46) consisting of patients attending the pain management unit who were not undertaking qigong. Only demographics and HLC information was collected from this group. Specific subgroups were compared to the control group using the unpaired *t*-test.

## 3. Results

### 3.1. General

The study consisted of *N* = 43 subjects, *N* = 29 in RIM1, and *N* = 29 in RIM2. In RIM2, 15 continued from RIM1, 11 were new to qigong, and 3 had prior qigong experience but did not participate in RIM1. Demographics are shown in Table 2. These are depicted as groups to align with quantitative data (first pair of columns) and as ongoing and new subjects to align with qualitative data and attitudinal analysis (second pair of columns). Participants were predominantly female, with mean age of 53–57 years and mean duration of chronic pain of 12–16 years. The most common forms of chronic pain were back pain and fibromyalgia. There were 4 discontinuations in RIM1 and 6 discontinuations in RIM2; these are not included in the reported numbers. There was no exact information collected, but contributing factors would be likely lack of effect, amount of effort involved, and other miscellaneous factors.

### 3.2. Quantitative Measures

**Figure 1:**
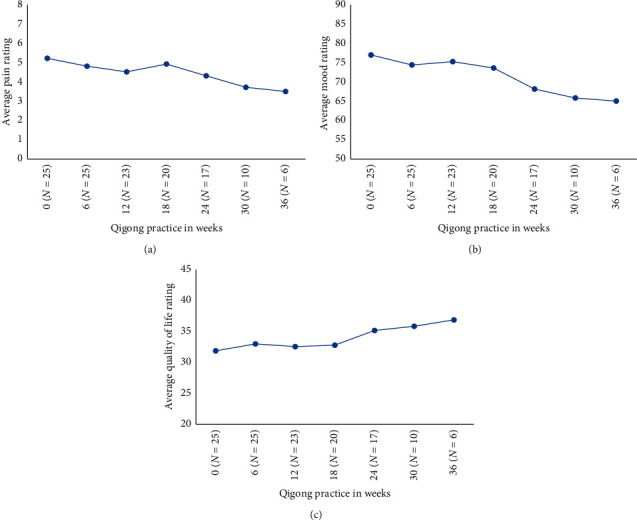
Average pain (a), average mood (b), and quality of life (c) from the RIM1 study start to 36 weeks of practice. Lower scores indicate less pain, less depression, and lower quality of life, respectively. Numbers below *x*-axis indicate those completing respective 6-week sessions. There were *N* = 29 participants in RIM1, but the 4 new subjects are included along with RIM2 new subjects to form a single group (see Figure 2). There were no significant longitudinal differences (*P* < 0.05 paired *t*-test) from the trial start (week 0) at any time.

Figure 1 presents quantitative measures for pain, mood, and quality of life Figures 1(a)–1(c) for RIM1 participants. It contains data for *N* = 25 ongoing subjects. The *N* = 4 new subjects from that cohort are included in the RIM2 data, which considers new subject experiences as a combined group (*N* = 15). There were no significant differences from entry values over the duration of the RIM1 trial.

**Figure 2:**
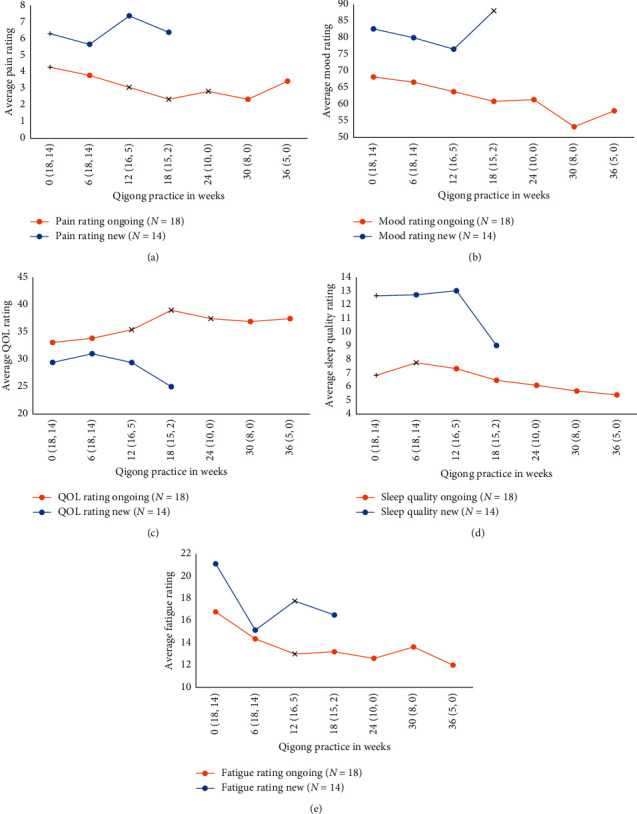
Average pain (a), average mood (b), quality of life (c), sleep quality (d), and fatigue (e) from the RIM2 study start to 36 weeks of practice. The orange line represents ongoing participants (*N* = 18) and the blue line new participants (*N* = 14). The *x*-axis shows the week of practice (# of ongoing, # of new). Lower scores indicate less pain, less depression, lower quality of life, improved sleep quality, and improved fatigue, respectively. Significant differences (*P* < 0.05) from the trial start (week 0) are indicated as a diagonal crossover of the data point. Start values differed significantly (*P* < 0.05, marked with a horizontal crossover of the data point) for average pain rating (a) and average sleep quality (d), with new participants experiencing worse pain and poorer sleep than the ongoing participants. No other measures differed between groups from RIM2 study start to 36 weeks of practice.

Figure 2 presents quantitative measures for pain, mood, quality of life Figures 2(a)–2(c), and sleep quality and fatigue Figures 2(d) and 2(e) for RIM2 participants. In the ongoing practice group, there were significant differences (*P* < 0.05) from entered values in pain (12–24 weeks), quality of life (12–24 weeks), sleep quality (6 weeks), and fatigue (12 weeks), with all changes reflecting symptom improvements except for sleep which was transiently worse. In those new to qigong, there were no significant changes from baselines.

### 3.3. Qualitative Comments

Table 3 presents qualitative comments for 10 subjects who practiced qigong for at least 3 years (RIM1, intervening year, RIM2). This group achieved good outcomes as revealed post hoc by the nature of their comments. Their experiences are in addition to *N* = 6 cases which are reported separately [14]. Overall, good or better outcomes were reported in 16/28 or 57% of long-term practitioners. While we use the term “good outcomes” to characterize these, many actually portray markedly improved function, including in those with chronic pain for decades who had previously accessed multiple conventional medical treatments. Most had practiced qigong for several years prior to RIM1, as noted in the table. Long-term qigong practitioners consistently reported benefits in pain, mood, sleep, and a reduced need for medications for these conditions. Additional benefits were noted in cardiovascular function (#14), asthma (#28), and psoriatic arthritis, diabetes, and bowel irregularities (#34). This group self-reported mean practice times of 78 to 118 min (∼1-2 hours) per day, daily or near-daily.

In the six cases reported separately [14], participants reported benefits in pain, mood and sleep, irritable bowel syndrome, food and environmental sensitivities, frozen shoulder, plantar fasciitis, head trauma, wrist tendonitis, immune function, respiratory function, sleep apnea, mobility, depression, anxiety, and posttraumatic stress. Most also commented on discontinuations of medications for pain and other conditions. Several commented directly on the profound nature of their health changes. This group self-reported extensive daily practice, with mean practice times of 99–190 min (∼1.5–3 hrs) per day, and consistent daily practice.

Table 4 presents qualitative comments for ongoing practitioners where outcomes were considered more variable based on a global assessment of comments (*N* = 13). Benefits in core domains were noted in several instances (#1, #8, #10, #19, #20, #38) and other health areas (#19 irritable bowel, #20 frozen shoulder). (This brings the total of those who experienced benefits from qigong practice to 22/28, or 78%.) Others reported little change or no benefit (#13, #25, #26). The variable group practiced for a mean of 25–38 min per day for 2–7 days per week. Some offered little in the way of comments (#4, #7, #24, #33) and reported minimal practice times (see footnote to Table 4).

Table 5 summarizes qualitative comments for those who were new to qigong when they commenced. One of these (#18) is included in Table 3 due to the nature of the outcomes and amount of practice. Most new participants attended for only a limited amount of time (1–3 sessions, 6–18 weeks) and reported minimal changes. A few noted some benefits in pain (#41, #46, #49, #52), while others comment on transient worsening of pain after classes (#39, #44, #50). New participants practiced for 34–47 min per day on 1–4 days per week.

Considered sequentially, qualitative comments published previously in a separate report [14] and in Tables 3–5 indicate a clear relationship between self-reported qigong practice times and health benefits in core and other domains. Those who practiced the most attained the most health gains. While there are imprecisions in the method of reporting practice times, even with this uncertainty, the relationship to outcomes is clear.

### 3.4. Attitudinal Assessments

Table 6 presents HLC scores for qigong subgroups categorized in different ways compared to control subjects attending the same facility (*N* = 46). Lower HLC scores indicate a stronger internal locus of control. All participants (*n* = 43) showed a lower HLC score compared to controls, as did ongoing qigong practitioners (*N* = 28). Those who were new to qigong (*N* = 15) trended lower compared to controls but were not significantly lower. Both those who attained good and variable outcomes were significantly lower than controls.

## 4. Discussion

This report documents the experiences of 43 people living with chronic pain (mean pain durations 12–15 years) who attended a tertiary-care pain management unit and undertook qigong as a self-care practice in addition to their usual medical care between 2014 and 2017. There is considerable heterogeneity in amounts of practice, both in the number of years of experience and in amounts of ongoing practice, and in outcomes as reflected in qualitative comments. Multiple viewpoints and subgroup considerations were required to derive meaningful information, and the dataset might best be considered as an extended case series. Pooled quantitative scores for pain, mood, and quality of life showed no longitudinal differences for ongoing qigong subjects during RIM1 but significant benefits during RIM2. With those new to qigong practice, there was little change in their scores. Results of the quantitative assessments are unremarkable and include many limitations in terms of standardized data collection and analysis methods. Of greater interest and importance in this study are qualitative comments related to the same domains, which provide a different view of health experiences, especially when considered as subgroups in relation to the amount of practice.

The experienced group represents those who have practiced qigong for >3 years (classes were offered each year; those continuing from RIM1 to RIM2 continued during the intervening, nonobserved year) and longer (up to 11 years). This information is of considerable value, as controlled trials for qigong in health areas relevant to chronic pain populations generally reflect durations of 6–24 weeks [3–5]. Collectively, qualitative comments consistently report benefits in core domains (pain, mood, sleep, quality of life) and multiple other diverse health benefits. Considered holistically, these outcomes can be characterized as good or very good. Some describe such profound health benefits that they can be considered remarkable and, for this reason, have been reported separately and in a more complete manner [14]. The self-reported practice times by these are considerable (1-2 hours/day near-daily or daily) (1.5–3 hours/day each day in case reports). Motivations for engaging in such extended practice times are the health benefits derived from the earlier practice and the wish to explore how far benefits can develop. In the subgroup where outcomes are variable, practice times were ∼0.5 hours/day for 2–7 days per week and are noticeably lesser than for those who reported good outcomes. Other qigong trials similarly report health benefits being related to the amount of practice within trials [9, 16] and between trials [4].

The seeming mismatch between quantitative and qualitative assessments for the ongoing practice group requires consideration. For these participants, entered values against which postpractice values are compared do not represent baselines due to the ongoing nature of their practice. In absolute terms, entered pain levels are in the moderate range and less than those in the new group and those in a larger trial of fibromyalgia subjects [9]. This factor applies to all quantitative measures. While mean entered values for ongoing participants compared to new participants reflect greater symptomatology in all instances for the new group (Figure 2), only the pain and sleep quality values differed significantly. Quantitative scores are widely used for evaluating pain outcomes, have been repeatedly validated, and provide important information for decision making. Nevertheless, the mismatch between the two approaches supports the need for pluralistic views on how to reflect health experiences of those with chronic pain [12, 13], especially when longer-term assessments are involved.

Those new to qigong must be considered separately, as their experiences with the practice differ vastly from others, and the aims of considering their outcomes differ. One new participant practiced diligently throughout, had good outcomes, and is included along with ongoing practitioners. Others attended only limited numbers of sessions and had lower daily and weekly practice times. Their outcomes are not as good as outcomes at 8–16 weeks in the controlled trial of this form of qigong in fibromyalgia [9]. The two groups had similar ages and durations of chronic pain. There are, however, differences in the method of delivery of instruction in these contexts. In the controlled trial, the training groups were all new to qigong at the same time, attended an orientation session that indicated health professional support, and received initial instruction in a more intensive manner (2 half-days, 8 hours). In the current setting, there was no orientation session, and new participants mixed with those who were experienced in weekly sessions (it can take 6–12 weeks before they are comfortable with techniques). Factors needed to encourage initiation and maintenance of practice need to be explored. Despite these considerations, it is important to recognize that some of the ongoing group in this report did encounter the practice through this delivery method, so it can be effective. (Others in the ongoing group encountered the practice through the trials, community-based practice, or prior exposure to pain self-management techniques.) The design of optimal program delivery requires careful consideration.

With regard to mechanisms by which health benefits occur, contemporary viewpoints consider endocrine, immune, and inflammatory biology mediation of mind-body therapies in general [19] and qigong in particular [20]. Recent neuroimaging of qigong in older adults indicates that practice affects brain regional and network activity [21], supporting earlier reports suggesting qigong modifies brain function [22–24]. A large trial involving functional magnetic resonance imaging of qigong in fibromyalgia subjects is currently underway (http://www.clinicaltrials.gov, NCT03890133) and should contribute to understanding neurobiological changes resulting from qigong practice in those with chronic pain. Chronic pain involves central sensitization (augmented signaling, diminished modulation) [25] and a shift in central pathways from nociceptive to emotional circuits [26, 27], and pain improvement reported by long-term qigong practitioners would likely be reflected in such changes. With inflammatory biology proposed as a key link in mind-body therapies [19], it is interesting to note that in those who practice qigong for 1–5 years (akin to experiences of some subjects in this study), there are alterations in the genomic expression of targets involved in the resolution of inflammation [28]. Furthermore, chronic pain is associated with disturbances in parasympathetic regulation [29], and as qigong promotes parasympathetic activity [30], this provides an additional potential mechanism for improvement to occur. Finally, the benefits of qigong in diverse health areas as noted by participants in this study, and especially in the six cases reported separately [14], indicate that qigong must restore integrative function and autoregulation, as elaborated in systems biology approaches [31].

The current study included an assessment of HLC. A more internal locus is associated with better outcomes with pain and depression [32, 33]. The qigong group held a more internal health locus compared to a control group. This is to be expected for a group that engages in a self-care practice, especially for such amounts of practice. All qigong subgroups differed from the control group, except those new to qigong simply showing a trend. It is not clear whether the loci of control scores change over time, but this could occur as a result of experience. Both control and qigong group HLC values in this study (means 36.07 and 31.79, respectively) are lower than those in other chronic pain studies using the same measure (fibromyalgia 45.73; rheumatoid arthritis 40.12) [18]; this may reflect differences in groups, cultures, or times. A more internal locus of control also is associated with the use of complementary therapies [34].

## 5. Conclusions

This report documents multiple health benefits over time in those with chronic pain who practice qigong, with benefits related to the amount of practice. Qualitative comments are essential for portraying these effects. Qigong can be characterized in several ways (see Introduction). The language of instruction can seem strange at times; it requires considerable diligence in practice, and it is not for everyone. However, given the profound nature of changes in subgroups described in this observational trial, further exploration of the potential for benefits in multiple health areas needs to occur. Both controlled trials over a specified interval and extension trials are needed, and with the latter approach especially, qualitative comments are essential to reflect experiences more completely.

## Figures and Tables

**Table 1:** Components of an observational trial of qigong as a voluntary self-care practice at a tertiary-care pain management unit.

Interval 1: RIM1	Interval 2: RIM2
July 1, 2014–May 31, 2015	July 1, 2016–May 31, 2017
Six 6-week sessions (summer, fall 1, fall 2, winter 1, winter 2, spring)	Six 6-week sessions (summer, fall 1, fall 2, winter 1, winter 2, spring)
Data collected at interval entry:	
(1) General: consent, demographics, medical history, qigong practice history	
(2) Quantitative measures^1^: BPI, POMS, SF-12, PSQI^2^, CFS^2^	
(3) Attitudinal measures: HLC	
Data collected at the end of each 6-week session:	
(1) Quantitative measures: BPI, POMS, SF-12, PSQI^2^, CFS^2^	
(2) Qualitative comments: open-ended survey containing questions relating to pain, sleep, other health areas, quality of life, current medication	
(3) Self-reported practice time: weekly log	
Number of participants: *N* = 29 (4 NEW to qigong)	Number of participants: *N* = 29 (11 NEW to qigong; *N* = 15 continued from RIM1; *N* = 3 with prior qigong experience joined RIM2)
Discontinuations: *N* = 4 (not included in total)	Discontinuations: *N* = 6 (not included in total)

BPI, Brief Pain Inventory; CFS, Chronic Fatigue Scale; HLC, Health Locus of Control; POMS, Profile of Mood Scores; PSQI, Pittsburgh Sleep Quality Index; SF-12 Quality of Life Survey. ^1^Quantitative measures at interval entry represent baselines only for those who were new to qigong; for those with prior experience, they represent anchor study entered values. ^2^Quantitative measures relating to sleep and fatigue were included in RIM2; other measures as in RIM1.

**Table 2:** Demographics for participants in two observational intervals as outlined in Table 1.

	RIM1 (*N* = 29)^1^	RIM2 (*N* = 29)^1^	Ongoing (*N* = 28)^1^	New (*N* = 15)^1^	Controls (*N* = 46)^2^
*Gender F:M: not stated*	18 : 11 : 0	22 : 6 : 1	13 : 15 : 0	0 : 14 : 1	37 : 9 : 0
Age, years (SD)	53.9 (10.7)	57.8 (12.1)	55.4 (10.6)	52.9 (14.6)	52.3 (12.3)
Duration of pain, years (SD)	15.0 (12.2)	14.8 (13.1)	14.8 (13.1)	12.3 (10.9)	—

*Qigong experience*					
None	4	11	0	15	—
6 weeks level 1 CFQ	3	1	3	0	
>6 weeks level 1 CFQ	8	5	9	0	
Levels 1 and 2 CFQ	13	12	15	15	
Unspecified	1	0	1	1	

*Pain diagnosis*					
Back pain	83%	48%	75%	73%	—
Fibromyalgia	48%	45%	50%	33%	
Headache	38%	31%	39%	20%	
Neuropathic pain	34%	31%	32%	40%	
Orofacial pain	24%	17%	21%	20%	
Osteoarthritis	21%	41%	21%	47%	
Cervical spine	17%	14%	18%	7%	
Rheumatoid arthritis	3%	10%	4%	0%	
Other	69%	72%	71%	67%	

CFQ, Chaoyi Fanhuan Qigong; COPD, chronic obstructive pulmonary disease; F, female; M, male; N, number; NSAIDs, nonsteroidal anti-inflammatory drugs. ^1^Total number of participants *N* = 43 (see Table 1 for disposition between RIM1 and RIM2). ^2^Controls were those with chronic pain attending the pain management unit but not undertaking qigong practice; only gender, age, and HLC information was collected (July-Aug of 2016).

**Table 3:** Qualitative comments in 10 participants who reported good outcomes during the observational trial^1^.

Participant number Gender (age, years) Pain diagnosis (years) (-) not stated	Practice amount^2^ days/week minutes/day # weeks	RIM1 comments 2014-2015 wks = weeks	RIM2 comments 2016-2017 wks = weeks
14 F (60) Back pain (22)	4/week 60–75/day 30 weeks RIM1	6 wks: doing qigong for years; more pain and worse sleep when not doing; 12 wks: myofascial pain flares helped by qigong; 30 wks: minimal flare-ups; greatly improved sleep; no longer needs meds [doing taxes and snow shoveling increase pain]	6 wks: great improvement in pain, coping skills and mood since CFQ 6+ years ago; blood pressure meds reduced and better maintained; cholesterol pills reduced; no longer need antidepressants, nausea or sleep meds; able to enjoy life so much more; so much better than before qigong; 12 wks: decreased pain meds and no longer need any for breakthrough pain; blood pressure meds reduced 75%; cholesterol pill reduced 50%; less angry and frustrated; 18-36 wks: (as before)^5^
	7/week 15–90/day 24 weeks RIM2		
	(started qigong in 2011)^3^		

17 F (62) Fibromyalgia (-)	7/week 200–240/day 30 weeks RIM1	6 wks: qigong has given me a life worth living (can walk, play; am calm, peaceful); improved overall health and outlook; 12 wks: no pain; after decades of pain and multiple health issues, years of trying many ways to improve, spending lots of time and money, now all I use is qigong for wellness; eyesight slightly improved; 24 wks: qigong helped so much; now have full, happy, peaceful life; chronic pain gone	—
	(started qigong in 2008)		

18 F (74) NEW^4^ Back pain (4) Osteoarthritis (10) Fibromyalgia (-) Plantar fasciitis (-)	5.5/week 50–70/day 24 weeks RIM1	6 wks: sleeping better; friends comment am looking better; gained weight (102 to 110 lbs); more relaxed; doing things not done before; 12 wks: see difference since qigong (weight); do not get pain relief in back, legs, feet at times; more energy; 18 wks: so pleased since qigong; able to things couldn't do before; some pain relief overall	6 wks: could never had been able to do the things I can today; much better [quality of life]; 12 wks: (no answer); 18 wks: no new changes; 24 wks: don't have as much pain in my back; have more energy and can certainly do more things; am much happier that I can go out with friends and not worry; stopped taking pantoprazole; 36 wks: (as before)
	5.5/week 40–55/day 24 weeks RIM2		
	(started qigong in 2014)		

22 F (67) Back pain (35) Osteoarthritis (35) Fibromyalgia (35)	7/week 45/day 24 weeks RIM1	6 wks: pain decreased since qigong (facet joint injections increased back pain for 10–14 days); sleep better; program has increased strength and helped with pain; 12 wks: much happier since qigong; better quality of life 90% of the time; 18 wks: doing good; sleep good since starting amitriptyline; 24 wks: don't feel so involved with pain, easier to cope	6 wks: pain reduced and more under control; able to function so much better; ups and downs but feel overall have made progress; [sleep] much better; not sleeping as well [heat, outdoor noise]; happier; feel much less moody; more in control of living my life; 12 wks: (as before); 18 wks: getting more sleep, sleeping better and more soundly; less pain throughout the night; more days with lesser pain; 24 wks: notice most improvements in sleep habits; 30 wks: (as before)
	7/week 35–55/day 36 weeks RIM2		
	(started qigong in 2010)		

27 M (63) Back pain (16) Neuropathic pain (10)	4.5/week 70–210/day 18 weeks RIM1	6 wks: this is hard; keeps my head clear; stronger inner core; 12 wks: qigong is saving my life; being with group is so important; qigong is very good pain relief; temper more controlled when doing qigong; 18 wks: without qigong life is pretty bad	—
	(started qigong in 2013)		

28 M (47) Back pain (10) Headache (14)	6.5/week 30–90/day 30 weeks RIM1	6 wks: pain relaxing back to normal; wellbeing improved; depression lifting; sleep returning to normal; 12 wks: easier to relax and unload pain; since qigong have stopped all pain meds; have not needed asthma meds in last year; 18 wks: pain dissipates much more quickly; sleep quality usually better; have stopped asthma and pain meds	—
	(started qigong in 2012)		

29 Not stated (31) Back pain (13)	7/week 150–180/day 24 weeks RIM1	6 wks: practiced qigong for 2 years; substantial improvements in that time; everything in my life has improved drastically because of the practice; sleep improved; using less and less cannabis; 18 wks: constant improvement for last 2 years; quality of life has improved exponentially	—
	(started qigong in 2012)		

30 F (54) Back pain (30) Cervical sprain (10)	7/week 50–60/day 24 weeks RIM1	6 wks: calmer at times and less depressed; sleep improved; 18 wks: very pleased with qigong; helped get me out of extreme chronic pain and keeps me going	6 wks: helps overall body, spirit, and mind; was in chronic pain and now no longer; returned to work; able to do housework; better sleeping; more comprehensive, more social, more activities; decrease in zantac; stopped nexium; decreased antidepressant; 12 wks: (as before); 18 wks: (as before); 36 wks: (as before)
	7/week 90–120/day 18 weeks RIM2		
	(started qigong in 2008)		

34 M (62) Chronic psoriatic arthritis (35)	— RIM1	—	6 wks: prior to CFQ, pain was like a knife slashing arms and legs 8-9/10; now pain free; improvements of psoriatic arthritis, diabetes, blood irregularities, mental health beyond what anticipated to be possible; overall quality of life greatly improved; improved ability to cope with health issues; no change medications; 12 wks: no pain; went from 500 mg naproxen twice daily 10+ years to no medication; insight into behavior patterns; more resilience; increased ability to calm self; less reactive; reduced insulin by ∼70%; decreased sleep disruption due to worry; more accepting and better able to settle; not confident that would still be alive if not for CFQ; increased relationship quality and quality of life; 18 wks: improvements most noticeable in ability to cope with stressors, awareness of attitudes/beliefs
	7/week 90/day 18 weeks RIM2		
	(started qigong in 2010)		

42 F (38) Fibromyalgia (2.5) Right shoulder/arm pain (7)	— RIM1	---	6 wks: my pain is significantly better; am able to do a lot more things; am not depressed anymore; sleep better; rarely wake up in the middle of the night; my life is much better since started CFQ; feel more confident; less stressed; stopped medication 1.5 year after starting qigong
	6/week 30/day 6 weeks RIM2		
	(started qigong in 2015)		

^1^Good outcomes were characterized post hoc based on the global qualitative comments relating to pain and other health areas. An additional *N* = 6 had notably good, even remarkable outcomes, and have been reported separately as a case series [14]. ^2^Mean (standard deviation) lower and upper range of practice times during sessions: 76(54) to 113(67) minutes/day, 4–7 days/week. ^3^Start dates for qigong practice stated for each participant; times range from 3 to 9 years. ^4^This participant was new to qigong in RIM1 and is included here due to the duration of practice and nature of outcomes. ^5^(as before) indicated by the participant or by recorder when no new information was offered at that interval.

**Table 4:** Qualitative comments by 13 participants who had variable outcomes during the observational trial^1^.

Participant number Gender (age, years) Diagnosis (years) (-) not stated	Amount of practice^2^ days/week minutes/day # weeks	RIM1 comments 2014-2015 wks = weeks	RIM2 comments 2016-2017 wks = weeks
1 F (56) Back pain (9) Neuropathic pain (9) Fibromyalgia (6)	5-6/week 35–40/day 12 weeks RIM1	6 wks: pain always less when do qigong regularly; sleep better but still take meds (less); people comment I look better; 12 wks: slip and fall on ice; leg spasms	6 wks: pain is more manageable; am less tense all the time; on lower dose of antidepressant and 200 mg less gabapentin; sleep is deeper and longer; am able to participate in family functions- before I usually cancelled or left early; 12 wks: (as before)^3^; 18 wks: (as before)
	6/week 30–60/day 18 weeks RIM2		

8 M (51) Back pain (30) Neuropathic pain (-) Headache (-)	5-6/week 15–45/day 24 weeks RIM1	6 wks: little better mood; sleeping bit better; feeling much better; 12 wks: easier to manage pain; somewhat better sleep; 18 wks: pain and discomfort constant; getting worse with age; tried everything; spinal fusion not fully fused since 1980s	—

10 M (64) Back pain (40) Headache, orofacial pain (40) Fibromyalgia (-)	4-5/week 10–20/day 6 weeks RIM1	6 wks: less shoulder pain; more relaxed	6 wks: no fear of spiders; less anxiety; 12 wks: (as before); 18 wks: not sure about change in pain level but helps to be in group with same people that deal with pain; sleeping a little better; able to fall asleep quicker; 24 wks: off non-prescription myoflex cream for pain; 30 wks: knee pain worse (not from qigong though); 36 wks: knee pain a bit better; instructor gave me advice to help knee pain
	3/week 15–20/day 36 weeks RIM2		

13 M (45) Neuropathic pain (11) Fibromyalgia (-)	2-3/week 10–20/day 18 weeks RIM1	6 wks: in pain after class; go home exhausted; 12 wks: pain in joints, pelvis, back; continuous pain, anxiety, depression; 18 wks: qigong seems to help but is exhausting	—

19 F (68) Back pain (6) Cervical sprain (6) Headache, orofacial pain (6) Fibromyalgia (14)	3/week 40–50/day 24 weeks RIM1	6 wks: pain and sleep improved with qigong; 12 wks: qigong makes it easier to cope; broken bite plate; increased jaw/neck/head pain; 18 wks: pain seems manageable if do qigong; new bite plate, decreased jaw/ear/neck pain; quality of life has improved; 24 wks: pain was decreased earlier but has now resumed, continuing with qigong	6 wks: pain certainly is more manageable; usually sleep better and have more energy; pain level reduced, overall wellbeing better and am more able to cope on daily basis; less migraines; able to do more with wrists (chopping etc.); CFQ helps to keep my irritable bowel on track; less tylenol and less headaches; 12 wks: not wearing bite plane at this time and haven't worn wrist splints for some time now; 36 weeks: not waking with nightmares as much; do not take any prescribed meds anymore, except synthroid
	5/week 35–45/day 36 weeks RIM2		

20 M (67) back pain (25)	2/week 15–30/day 24 weeks RIM1	6 wks: when practice regulatory, less pain; with flare-up, immediate pain reduction with qigong; sleep better; when wake in pain, do (qigong) movements to eliminate pain and go back to sleep; 12 wks: been practicing since 2011 and overall, quality of life and outlook are much improved; 18 wks: continue to have positive outlook knowing that the practice works; 24 wks: when I practice “regularly”, less pain and discomfort - when don't, as in past month, have increased pain and stiffness	6 wks: had a frozen shoulder for 30+ years and now have no symptoms; when do regular practice, my hip pain reduced noticeably; [sleep] better with regular practice; 12 wks (as before)
	3/week 15–30/day 12 weeks RIM2		

25 M (30) Back pain (16) Neuropathic pain (16) Headache (14) Fibromyalgia (16)	7/week 40–50/day 24 weeks RIM1	6 wks: helped to move and stretch, still same pain; healthier and more relaxed; 12 wks: quality of life still on downside; 18 wks: pain staying level but mobility improving; 24 wks: sleep still restless and rare	—

26 M (55) Back pain (3.5) Osteoarthritis (3.5)	4/week 30–40/day 12 weeks RIM1	6 wks: tried pain meds and many hours of alternative therapies; pain meds cause too many side effects with no pain relief; others give temporary/partial relief only; doing qigong since 2014 and although it helps tolerate pain, still have lots of pain; 12 wks: increased stress [elderly parent] has impacted pain and limited practice	—

38 M (81) Osteoarthritis (10)	—	—	6 wks: CFQ has provided positive mental awareness of potential to heal within myself; recently seemed to be releasing pain and muscle distress from more than current injury in last 6 years; [pain] reduced by 50%; have incorporated a wellness program and have improved my physical wellness by at least 100%; wellness/emotional wellness have improved; no change in medications
	6/week 40/day 6 weeks RIM2		

^1^Outcomes were characterized as variable based on a global assessment of comments. A further *N* = 4 participants (#4, #7, #24, #33) offered little in the way of qualitative comments (indicating minimal pain, no change, less pain and better sleep, qigong not helping, respectively); these generally reported practicing 15–30 min/day, 3–5 days/week for 1–4 sessions. ^2^Mean (standard deviation) lower to upper range of practice times during sessions: 25(12) to 38(12) minutes/day, 2–7 days/week. ^3^(as before) indicated by participant, or by recorder when no new information offered at interval.

**Table 5:** Qualitative comments by *N* = 14 who were new to qigong practice on entering the observational trial^1^.

Participant number Gender (age, years) Pain diagnosis (years) (- not stated)	Practice amount^2^ days/week minutes/day # weeks	RIM2 comments 2016-2017 wks = weeks
46 not given (26)^3^ Back pain (9) Fibromyalgia (1)	4/week 30–90/day 18 weeks RIM2	6 wks: [pain] improvement; fewer full body flare-ups; localized flare-ups resolve more quickly; calmer; easier to identify pain/stress triggers; fatigue hasn't been quite as bad; calmer before bed; feel rested sometimes; less stress, better able to deal with stress; able to do slightly more than usual; need to rest (bed-ridden) little less often; 12 wks: less [pain] overall; muscles feel like they're learning to relax; overall decrease in tension, stiffness, and soreness; levels and sleep have improved; decreased panic, anxiety, and depression; waking earlier with a feeling of rest; better able to do activities and to enjoy those activities; nortriptyline decreased from 20 to 10 mg because of qigong. 18 wks: anxiety and depression both lessened; feeling calmer; overall improvements in mental clarity and in sense of self

37 F (80) Back pain (-) Orofacial pain (-) Osteoarthritis (-)	2/week 45/day 6 weeks RIM2	6 wks: CFQ has improved by balance; feel relaxed and more settled after a class; pain has remained consistent depending on my physical activity; no change [in] medications

39 F (32) Back pain (4) Neuropathic pain (4)	1/week 60/day 6 weeks RIM2	6 wks: [pain spikes] during class and for a day or so afterward; feel more in control of my pain; no change in medications

41 F (55) Back pain (-) Orofacial pain (38) Osteoarthritis (40) Fibromyalgia (40)	1.5/week 15–45/day 12 weeks RIM2	6 wks: decrease in joint pain; not waking up as many times; no change medications; 12 wks: some change in pain and energy levels; think gallbladder recovered quicker with CFQ exercises; am happy to find an exercise program that doesn't leave me feeling worse after; this gives me energy and helps lift spirits

44 F (50) Back pain (9) Cervical sprain (4) Neuropathic pain (9) Headache (9)	4/week 30/day 12 weeks RIM2	6 wks: have new pain area after CFQ but subsides a few hours after session; increased fatigue post session, returns to normal fatigue day 3 post session; do feel a little more peaceful; no sleep changes; do quick bit of the program to relax before bed; helps clear my mind; a little more social; 12 wks: increased pain and soreness; increased fatigue; some decrease in muscle tension; mentally feel better; positivity and hope for improvement; decreased Flexeril and substituted [morning] dose with Tylenol XS for same result

47 F (60) Neuropathic pain (12.5)	3/week 20/day 6 weeks RIM2	6 wks: from the first class have seen improvement; [quality of life] has increased significantly compared to where was before

48 F (71) Neuropathic pain (3) Osteoarthritis (20)	2-3/week 15–45/day 18 weeks RIM2	6 wks: (no comments); 12 wks: pain a little better when I do [qigong]; feel somewhat better; no medication changes; 18 wks: no real changes [in pain] but helping a little bit

49 F (47) Back pain (10) Headache (-) Fibromyalgia (30)	3-4/week 65–70/day 6 weeks RIM2	6 wks: the more I do, the better I feel; less stiff and sore; more energy; still having problems sleeping; more practice at home may help with this; get more done in a day

50 F (47) Osteoarthritis (20) Fibromyalgia (4)	2.5–3/week 15/day 12 weeks RIM2	6 wks: increased pain in right side and heaviness the day after sessions, which subsides and I feel better; more positive when in the sessions; being part of the CFQ program has been a blessing, it has given me something positive to look forward to; 12 wks: more pain in my hands, wrists, ankles and toes; more fatigued during this 6-week session; may also be due to increased activity this session; sleep has been deeper at times; feel very energized after each session; more positive about daily activities

52 F (60) Back pain (20) Osteoarthritis (20)	4/week 30/day 6 weeks RIM2	6 wks: helps my back [pain] quite a bit, [but not my] neck, legs, and feet; [can] walk a little longer; no medication changes

^1^ *N* = 4 participants (#6, #21, #31 in RIM1, #51 in RIM2) offered minimal comments (e.g., no change, too early to tell) in the qualitative sections; these reported practicing as recommended, but for only one 6-week session. ^2^Mean (standard deviation) lower and upper range of practice times for those in table: 33(18) to 45(22) minutes/day, 1–5.5 days/week. ^3^Good outcome; moved forward in table so that it is presented as the first case.

**Table 6:** Health Locus of Control (HLC) scores for those undertaking qigong practice as a voluntary self-care practice at a Pain Management Unit.

	Controls *N* = 46	Qigong groups				
		All *N* = 43 *P* vs. controls	Ongoing *N* = 28^2^ *P* vs. controls	New *N* = 15 *P* vs. controls	*N* = 15 good outcomes^2^ *P* vs. controls	*N* = 13 variable outcomes *P* vs. controls
Health Locus of Control (HLC)^1^	36.07 ± 6.98	31.79 ± 6.59 *P* < 0.05	30.89 ± 6.85 *P* < 0.05	33.47 ± 5.94 NS	30.73 ± 7.83 *P* < 0.05	31.08 ± 5.82 *P* < 0.05

All values in the table are mean ± standard deviation. *P* values compared to controls are by the unpaired t-test. NS indicates not significantly different, *P* > 0.05. ^1^The HLC scale consists of 11 items, each scored on a 6-point scale (1 = strongly disagree, 2 = moderately disagree, 3 = somewhat disagree, 4 = somewhat agree, 5 = moderately agree, 6 = strongly agree). (1) If I take care of myself, I can avoid illness. (2) Whenever I get sick it is because of something I've done or not done. (3) Good health is largely a matter of good fortune. (4) No matter what I do, if I am going to get sick I will get sick. (5) Most people do not realize the extent to which their illnesses are controlled by accidental happenings. (6) I can only do what my doctor tells me to do. (7) There are so many strange diseases around that you can never know how or when you might pick them up. (8) When I feel ill, I know it is because I have not been getting the proper exercise or eating right. (9) People who never get sick are just plain lucky. (10) People's ill health results from their own carelessness. (11) I am fairly responsible for my health. *Note*. Items 1,2,8,10,11 are reverse-scored. An internal locus of control is indicated by lower composite scores, and an external locus by higher composite scores. (Scale from Wallston et al. )^18^. ^2^ONGOING and GOOD OUTCOME groups represent quantitative data for *N* = 6 reported in a case report^15^ and *N* = 9 good outcomes in Table 3. The one subject who was new to qigong at the start of RIM1 included in Table 3 remains in the NEW group for the attitudinal analysis.

## Data Availability

Data are available upon request to the principal investigator.

## Abbreviations

BPI: Brief Pain Inventory
CFQ: Chaoyi Fanhuan Qigong
CFS: Chronic Fatigue Scale
COPD: Chronic obstructive pulmonary disease
F: Female
HLC: Health locus of control
M: Male
N: Number
NSAIDs: Nonsteroidal anti-inflammatory drugs
POMS: Profile of Mood Scores
PSQI: Pittsburgh Sleep Quality Index
SF-12: Quality of Life Survey.
